# RISK AND PROTECTIVE FACTORS FOR INCREASING ISOLATION AND LONELINESS AMONG OLDER ADULTS

**DOI:** 10.1093/geroni/igae098.2542

**Published:** 2024-12-31

**Authors:** Tiffany Hughes, Fang Fang, Yueting Wang, Erin Jacobsen

**Affiliations:** Midwestern University, Glendale, Arizona, United States; Old Dominion University-EVMS, Norfolk, Virginia, United States; University of Pittsburgh, Pittsburgh, Pennsylvania, United States; University of Pittsburgh, Pittsburgh, Pennsylvania, United States

## Abstract

Social isolation and loneliness are associated with poor health and quality of life as well as decreased longevity. This study examines patterns of change in self-reported isolation and loneliness, and factors associated with these patterns, using data from 1,474 cognitively normal participants followed annually for up to 12 years in a population-based cohort study of older adults. Composite measures of isolation and loneliness were created, and trajectory analyses were conducted to detect patterns of change in these composites over time. Multinomial logistic regression tested associations of baseline demographic, health, and lifestyle factors with patterns of change in isolation and loneliness. Trajectory analyses revealed three patterns of change in isolation - slowly decreasing, slowly increasing, and quickly increasing; and three patterns of change in loneliness - stable, increasing, and decreasing. Participants who had ever smoked, currently drank alcohol, were physically active, had hypertension, used the computer, and were not obese at baseline were less likely to be in the quickly increasing isolation pattern. Those with greater isolation and loneliness at baseline, who lived alone, were obese, did not have daytime sleepiness, and rated health as very good or excellent were more likely to be in the increasing loneliness pattern. Distinct patterns of change in isolation and loneliness were found over 12 years with different factors associated with risk for changing isolation and loneliness over time. Understanding characteristics of participants associated with change in isolation and loneliness over time may help to identify older adults for interventions.

